# Design and Validation of Android Smartphone Based Wireless Structural Vibration Monitoring System

**DOI:** 10.3390/s20174799

**Published:** 2020-08-25

**Authors:** Dongyu Zhang, Jiadong Tian, Hui Li

**Affiliations:** 1 Key Lab of Structures Dynamic Behavior and Control (Harbin Institute of Technology), Ministry of Education, Harbin 150090, China; tianjiadong@stu.hit.edu.cn (J.T.); lihui@hit.edu.cn (H.L.); 2 School of Civil Engineering, Harbin Institute of Technology, Harbin 150090, China

**Keywords:** smartphone, wireless sensor network, time synchronization, structural vibration monitoring

## Abstract

Vibration monitoring is one of crucial functions of structural health monitoring (SHM) systems. Traditional structural vibration monitoring usually relies on specialized sensors, data transmission and acquisition equipment, which are expensive and may not be easily available in urgently needed situations like post-disaster structural evaluation. Therefore, developing an affordable and efficient structural vibration monitoring technique becomes an important topic in SHM research. In this paper, the authors developed an android system APP that can easily convert multiple android smartphones into a wireless structural vibration monitoring system. To make the designed system reliable and easy to use, the server/client architecture is adopted. One smartphone is designated as the serve of the system to remotely control all other smartphones, which function as sensors to measure structural vibration. An efficient method is proposed herein to establish the smartphone-based structural vibration monitoring network, allowing the server smartphone to quickly and easily connect multiple sensor smartphones to form the wireless network for structural vibration monitoring. Additionally, a synchronization method is also proposed to synchronize different smartphones for simultaneously measuring structural vibration. To verify the time synchronization accuracy of the developed system, an experiment is designed and conducted. Moreover, a new analysis method of the time synchronization accuracy is also proposed, which verifies that the designed smartphone-based monitoring can achieve the millisecond-level time synchronization accuracy. Finally, a shaking table experiment is conducted on a three-story bench-scale structural model, the results of which demonstrate that the designed smartphone-based wireless structural vibration monitoring system can quite accurately identify the modal parameters of the tested structure.

## 1. Introduction

With gradual degradation of many important civil structures (e.g., high-rise buildings and long-span bridges), the safety of these structures becomes a public concern, particularly after these structures are subjected to extreme loads (e.g., earthquakes or strong winds). Structural health monitoring (SHM) systems are an effective measure to detect structural damage in time and ensure the safe operation of these engineering structures [1,2,3]. Structural vibration monitoring is one of core functions of SHM systems. Traditional structural vibration monitoring generally relies on expensive specialized sensors, data transmission and acquisition equipment. Thus, only very important structures are affordable to install a SHM system. However, when an extreme event (e.g., earthquake) occurs, many structures may be damaged. Since it is impractical to install SHM systems on all structures in advance, it will be of great value to develop new affordable and efficient structural vibration monitoring technique, which can be easily available and quickly deployed to assess the structural integrity and safety after the extreme events.

In recent years, smartphone technology has advanced very rapidly. Nowadays, smartphones are not only equipped with powerful data acquisition, processing, storage and transmission capabilities but also integrated with a variety of high-performance vibration sensors (e.g., accelerometers and gyroscopes), which makes it possible to utilize smartphones to carry out the SHM tasks, like structural vibration monitoring.

Compared with the traditional dedicated SHM system, using smartphones for structural vibration monitoring has many attractive advantages: (1) smartphones are very popular and easily available; thus, it is possible to quickly deploy a large number of smartphones as vibration sensors on the structure at relatively low cost, which may be very useful in emergent case, like post-disaster structural evaluation, to quickly access structural integrity condition; (2) smartphones are integrated with a lot of telecommunication technology (e.g., WIFI, Bluetooth, near-field communication and 3G/4G etc.), which provides many flexible ways to build a smartphone-based wireless structural health monitoring network, allowing the smartphones to easily communicate and transmit the monitoring data one other; (3) smartphones also have powerful computing and data storing capacity, which can be used to analyze the monitored structural responses and evaluate structural health status directly on the smartphones, achieving the efficient distributed SHM.

Due to the above attracting features of using smartphones to carry out SHM tasks, many researchers have conducted different research on smartphone-based SHM applications. Kong etc. [4] verified the possibility of using accelerometers inside smartphones to measure the building accelerations, which were excited by a shake installed on the roof. Feng et al. [5] proposed to use ubiquitous smartphones to form a low-cost wireless citizen sensor network, measuring structural vibrations during earthquakes to facilitate post-disaster structural evaluation. Ozer et al. [6] investigated to use heterogeneous sensor data in iPhone to obtain instantaneous phone’s orientation, which can be utilized to correct misaligned sensor signals and improve the efficiency of structural data analysis. Zhao et al. [7] investigated the possibility of using smartphones to carry out dynamic testing of cable structures. Li et al. [8] utilized the camera of smartphone to measure the interstory drift of building structures during earthquakes. Xie et al. [9] conducted an experiment to verify the feasibility of using smartphones to perform structural damage detection of a 3-D steel frame structure. Matarazzo et al. [10] and Ozer et al. [11] proposed to use smartphones to identify the bridge’s modal frequencies, which provides a low-cost and efficient method to collect the structural health condition data of the bridges.

Despite a lot of smartphone-based SHM research have been carried out, there are still many challenges for smartphone-based SHM. First, the smartphone-integrated vibration sensor is not of scientific instrument standard and the sensors in different smartphone may have large different accuracy, which greatly affect the quality of the measured data. Second, since smartphones are not initially designed as sensors, they cannot collect signals from integrated sensors according to a strict time schedule as regular sensors do, which poses a difficulty for data analysis. Third, the sampling rate of the smartphone sensors may not be higher enough to measure the vibration of a very stiff structure, which limits the application ranges of the smartphone-based SHM.

Moreover, extensive research of using smartphones for SHM have been conducted, but most of them focus on the utilization of single smartphone. Although there are a few studies [11,12,13] that used multiple smartphones together, the measurement data from these smartphones are not time-synchronized. Therefore, all structural analyses that requires the synchronized measurements cannot be applied. For structural modal parameter identification, many methods (e.g., frequency domain decomposition, Next-ERA, subspace identification, etc.) need the measured structural responses to be synchronized; several researches showed [14,15,16] that large asynchronous error may significantly deteriorate the accuracy of identified structural modal parameters. Hence, developing techniques that can efficiently organize multiple smartphones to work coordinately and simultaneously measure structural responses with high accuracy of time synchronization will be of great value for the SHM applications like structural vibration monitoring.

In this paper, an android system-based APP, developed by the authors, is presented, which can easily convert multiple android smartphones into a wireless structural vibration monitoring system, measuring structural vibration with high accuracy of time synchronization. The developed smartphone-based system provides a new and easy way to quickly deploy a wireless structural vibration monitoring network on the structure, which is of great value for the applications like post-disaster structural evaluation.

The organization of the paper is listed as follows. First, the system architecture of the smartphone-based system is introduced in detail, which can efficiently organize multiple smartphones to form a wireless sensor monitoring network. Second, a time synchronizing method is developed for the smartphone-based system, which can synchronize the time clocks of the smartphones with high accuracy for simultaneously structural vibration monitoring. Then, an experiment is designed and conducted to verify the time synchronization accuracy of the designed smartphone-based monitoring system; and a new upsampling-based analysis method is also proposed to improve the resolution of time synchronization accuracy analysis. Finally, shake table tests are conducted on a three-story small-scale structural model to check the performance of the developed smartphone-based monitoring system for structural modal parameter identification, demonstrating that the system can quite accurately identify the structural natural frequencies and mode shapes.

## 2. Development of Smartphone-Based Wireless Structural Vibration Monitoring System

### 2.1. System Composition

**Figure 1:**
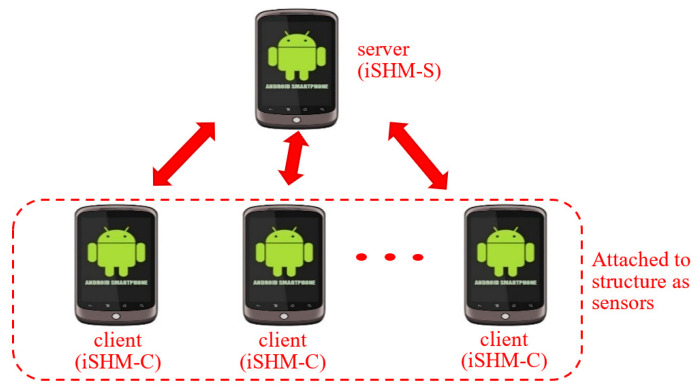
Network architecture of smartphone-based structural vibration monitoring system.

To convert multiple smartphones into a wireless sensor network for structural vibration monitoring, an android system-based APP was developed by the authors. The APP adopts the server/client architecture and consists of two parts, namely iSHM-Server (iSHM-S) and iSHM-Client (iSHM-C). One smartphone installing the iSHM-S, called server smartphone, serves as the server of the wireless monitoring network. The other smartphones installing the iSHM-C, called sensor smartphones, work as sensor nodes of the wireless network to measure structural vibrations. The users can utilize the server smartphone to remotely control all sensor smartphones to conduct all kinds of operations for structural vibration monitoring (e.g., time synchronization of wireless sensors, structural vibration measurement, etc.). The system architecture of the smartphone-based structural vibration system is shown Figure 1.

**Figure 2:**
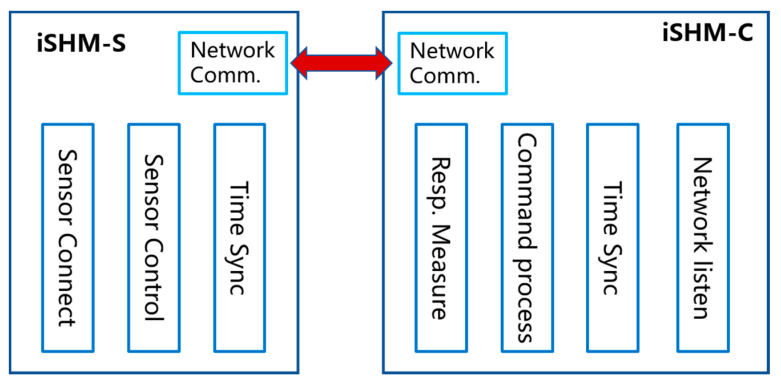
Functional modules of iSHM-S and iSHM-C.

To facilitate the maintenance and future development of the APP, all functions of the APP are modular in design, as shown in Figure 2. The iSHM-S includes three modules: Sensor connection, sensor control, time synchronization. The sensor connection module is responsible for setting up a local WIFI network, establishing the network connections between the server sand sensor smartphones. The sensor control module can accept the user’s commands to fully control the operation of sensor smartphones. The time synchronization module is to synchronize the local time clocks of the server and sensor smartphones, allowing all sensor smartphones to simultaneously measure structural responses. Similarly, the iSHM-C consists of four modules: network listening, command process, time synchronization, response measurement. The network listening module is used to listen the command sent by the server smartphone. The command process is responsible for parsing the server smartphone’s command and carrying out corresponding operations. The time synchronization module is to synchronize the local time clocks of sensor smartphones with respect to that of server smartphone. The response measurement module is to measure and record the structural vibration data.

### 2.2. Selection of Wireless Communication Technology

To build a smartphone-based wireless monitoring system, the first step is to select the wireless communication technology that the system is used to connect different sensor nodes. Generally, smartphones integrate several wireless communication technologies, including WIFI, 4G, Bluetooth, near field communication (NFC). Table 1 lists the important technical parameters (e.g., number of connected devices, connection distance, bandwidth, etc.) of these wireless communication technologies. After comparing these parameters, it becomes clear that WIFI is the most suitable technology for the smartphone-based structural vibration monitoring system to be developed.

### 2.3. Operation Procedure of System

In order for the server and sensor smartphones to communicate each other, creating a reliable network connection between them is a critical step, which requires that the IP addresses of the server and sensor smartphones must be known by the other. However, since the smartphone is not initially designed as a wireless sensor node, its IP address is not fixed and generally unknown in advance. Although the IP address of server smartphone can be manually input into all sensor smartphones, it requires a lot of human intervention, making the designed system difficult to use.

To solve the above difficulty as well as make the network connection convenient and secure, the Android AP network was adopted herein. When the iSHM-S is started, it will use the Android AP network to create a local WIFI network (hotspot), through which the other sensor smartphones can connect. The Android AP network will automatically assign the IP addresses to the connected sensor smartphones, which are saved in the system cache file of Android system on the server smartphone. The iSHM-S will read the system cache file to obtain the sensor smartphones’ IP addresses. Then, the iSHM-S utilizes the Android API Socket to initiate a dedicated network connection with each sensor smartphone via TCP/IP protocol, through which the server smartphone can remotely control all sensor smartphones to conduct the operations of time synchronization, structural vibration measuring, measurement data storage and management, etc. The above procedure is very efficient to setup a smartphone-based structural vibration monitoring network. It only takes about 20 s to set up one sensor smartphone, greatly facilitating the quick deployment of a large number of sensor smartphones on the structure.

**Figure 3:**
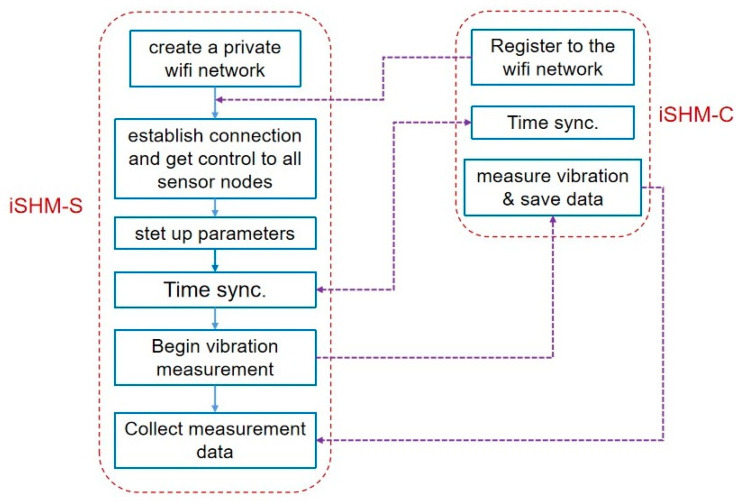
Operation procedure of iSHM-S and iSHM-C.

The full operation procedure of the iSHM-S and iSHM-C is illustrated in Figure 3. First, the server smartphone creates a private WIFI network, through which all sensor smartphones register to connect. Second, the server smartphone uses the IP information of the connected sensor smartphones to establish a dedicated TCP/IP connection for each of sensor smartphones to remotely control them. Next, the time synchronization operation, proposed in Section 3, is conducted to obtain the time differences of the local time clocks between the server smartphone and every sensor smartphone. After the time synchronization is finished, the server smartphone will send ‘start measuring’ command to all sensor smartphones, and the sensor smartphones will then begin to collect structural responses based on their own local time clocks. After all sensor smartphones finished measuring structural responses, they will send the measurement data file with the local time stamps back to the server smartphone, on which the measurement data of all sensor smartphones are synchronized to a uniform time clock based on the previous result of the time synchronization operation.

### 2.4. Graphic User Interface (GUI)

**Figure 4:**
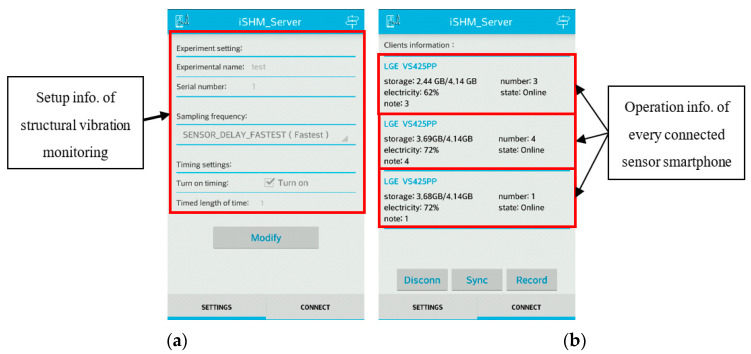
Graphic user interface (GUI) of iSHM-S: (**a**) setting page; (**b**) connect page.

**Figure 5:**
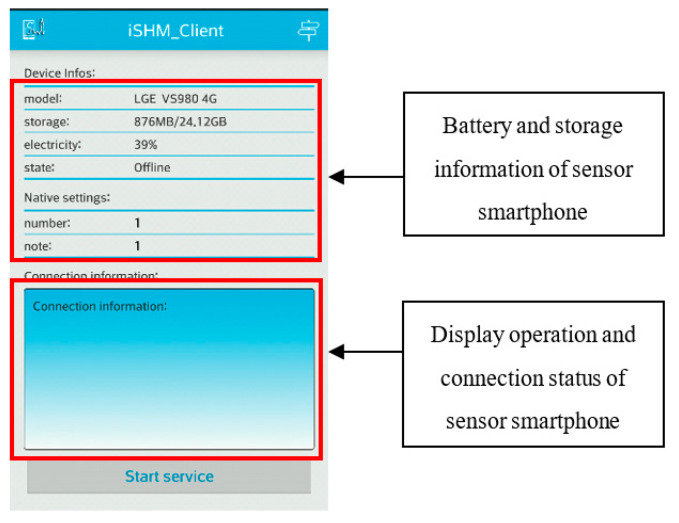
GUI of iSHM-C.

To make the designed smartphone-based monitoring system easy to use, the APP’s graphic user interfaces (GUI) is designed to be concise and easily understandable. Figure 4a shows the GUI of the setting page in the iSHM-S, in which the name and number of the vibration test as well as the sampling rate and the duration length of the measurements can be set up by the user. Figure 4b shows the GUI of the connect page in the iSHM-S after all sensor smartphones have been successfully connected to the server smartphone; in this page, the detailed information, like the sensor node number, the remaining electricity and storage capacity, etc., of all connected sensor smartphones are displayed, which allows the user to easily keep track of the operational status of every sensor smartphone. Figure 5 shows the GUI of the iSHM-C, which provides the information about operation status of sensor smartphone, including the phone number, remaining electricity power and storage capacity as well as connection status of the sensor smartphone.

## 3. Time Synchronization of Smartphone-Based Monitoring System

For a structural vibration measurement system, time synchronization among different sensors is of great importance, because asynchronized structural measurements may lead to large errors of structural modal parameter estimation [14,15,16]. In contrast to the wired SHM system, in which all sensors are securely connected via electrical cables and can synchronously collect data based on the uniform time clock of the data acquisition system, every wireless sensor collect data according to its own local time clock. Therefore, wireless monitoring systems have to adopt some kinds of time synchronization algorithms to keep the local time clocks of all wireless sensors synchronized.

There are many time synchronization algorithms for wireless sensor network [17]. In this study, according to the characteristics of the designed smartphone-based wireless system, a sender-receiver based time synchronization algorithms is deployed, which is explained thereafter.

**Figure 6:**
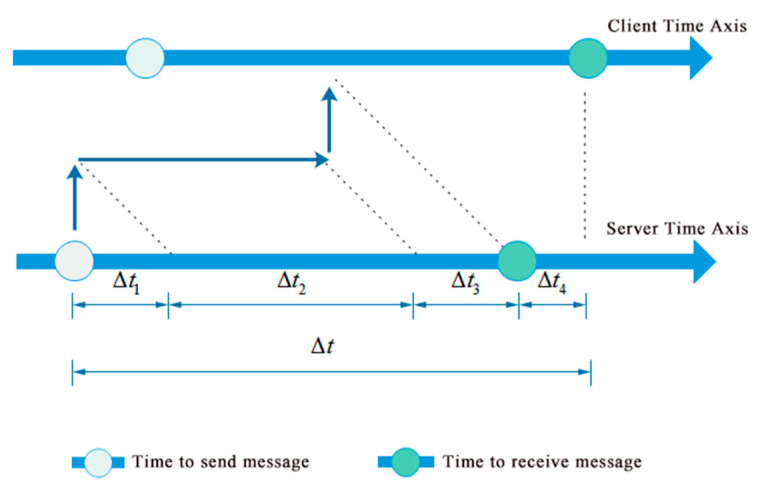
Time analysis of a message transfer from server to client.

Figure 6 shows the process of a message that is sent from the server (i.e., server smartphone) to the client (i.e., sensor smartphone). Then, the time delay Δt between the time when the message is sent in the server and the time when the message is received by the client can be calculated by Equation (1).

(1) $$\Delta t=\Delta t_{1}+\Delta t_{2}+\Delta t_{3}+\Delta t_{4}$$

where $\Delta t_{1}$ is the time delay that the server needs to send out the message, $\Delta t_{2}$ is the time delay of network communication, $\Delta t_{3}$ is the time delay that the client needs to process the message, $\Delta t_{4}$ is the difference of local time clock between the server and the client, which needs determining to synchronize the local time clocks of the server and the client. Since $\Delta t_{1}$, $\Delta t_{2}$ and $\Delta t_{3}$ in Equation (1) are related to the operation status of the server and client as well as the congestion condition of network communication, which are unknown in advance, $\Delta t_{4}$ cannot be directly determined by Equation (1).

**Figure 7:**
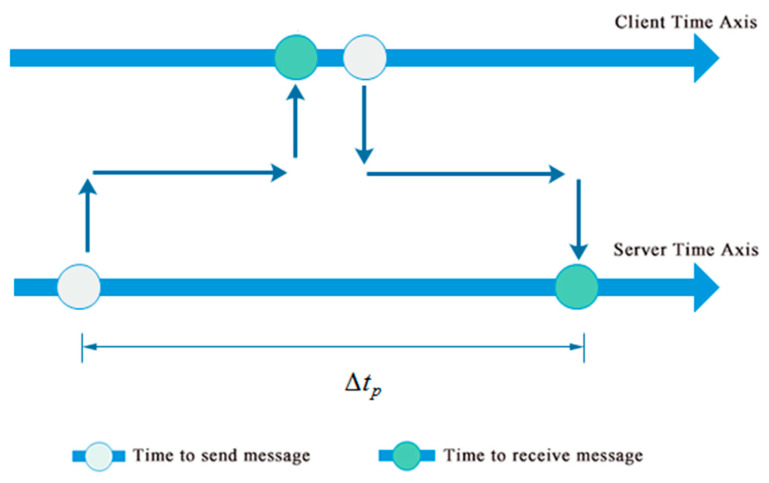
Time analysis of ping command sent by server.

To obtain the local time difference $\Delta t_{4}$, the following operations are carried out. First, the server sends a package with the server’s time stamp, following with a ping command to the client. After the client receives the package, it will calculate the time difference Δt based on the local time of receiving the message, and send this value back to the server. Simultaneously, the client will automatically reply to the ping command of the server. As shown in Figure 7, the time difference $\Delta t_{p}$ between the time when the server sends out ping command and the time when the server receives the reply from the client can be approximated by Equation (2).

(2) $$\Delta t_{p}\approx 2\left(\Delta t_{1}+\Delta t_{2}+\Delta t_{3}\right)$$

Since the client will immediately reply to the ping command from the server, the time delay between the client’s receiving and replying the ping command is ignored herein. Combining Equations (1) and (2), the local time difference $\Delta t_{4}$ can be estimated as:

(3) $$\Delta t_{4}\approx \Delta t-\frac{\Delta t_{p}}{2}$$

**Figure 8:**
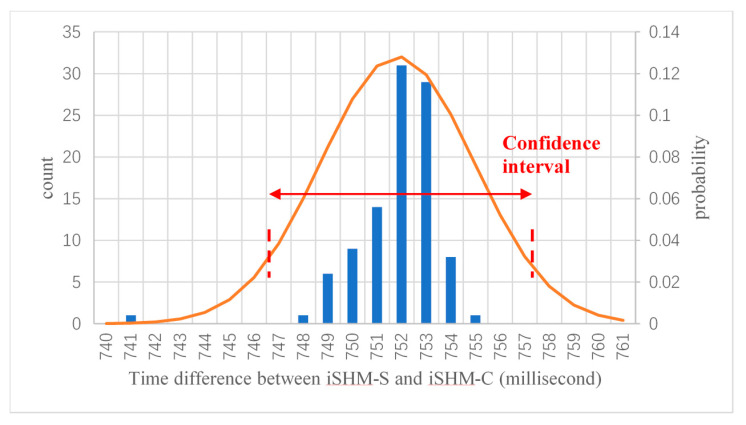
Distribution of estimated time differences $\Delta t_{4}$ of 100-time synchronization tests.

However, due to the uncertainties in the wireless communication as well as the operation status of the server and client, the estimated time differences $\Delta t_{4}$ will be varying in different tests. For example, Figure 8 demonstrates the distribution of the estimated time differences $\Delta t_{4}$ of 100 synchronization tests, which has a standard deviation of 3.1 millisecond. To further reduce the uncertainty in the time synchronization, the above message-sending procedure is carried out *n* times for each time synchronization operation, and the mean value of all *n* estimated time differences $\Delta t_{4}$ in Equation (3) will be used to synchronize the local time clocks of the server and the client.

The reason that the above averaging operation can reduce the uncertainty of time synchronization is explained as follows. It is assumed herein that all the estimated time differences are statistically independent and identically distributed, which follow a normal probability distribution N(μ,σ), where μ is the mean and assumed to be the true time difference between the server and the client; σ is the standard deviation of the distribution. Based on the above assumption, it can be easily obtained that the standard deviation of the mean estimate of the *n* results will reduce to $\sigma /\sqrt{n}$, and the 95% confident range of the mean estimate is $\left[\mu -1.95\sigma /\sqrt{n},\mu +1.95\sigma /\sqrt{n}\right]$. Clearly, increasing the average time *n* can lead to a more accurate time synchronization result. After considering the trade-off between the synchronization accuracy and the synchronization operation time, the average time *n* is selected as 30 in the APP design; consequently, the time synchronization accuracy can reach around one millisecond. It is noted that the above time synchronization analysis is conducted based on a very idealistic assumption, which may not be true in real situation. Thus, an experiment will be performed in the next section to verify the effectiveness of the proposed time synchronization method for the developed smartphone-based structural vibration monitoring system.

## 4. Experimental Verification of Time Synchronization Accuracy

In Section 3, a time synchronization method is proposed for the smartphone-based structural vibration monitoring system, which theoretically can achieve time synchronization accuracy of millisecond level. To check the time synchronization accuracy that the designed smartphone-based structural vibration monitoring system can actually achieve, an experiment is designed and conducted in this section.

### 4.1. Experiment Design

To evaluate the time synchronization accuracy, the local time clocks of both server and sensor smartphones are needed. However, since smartphones are not initially designed as wireless sensors, it is very difficult to simultaneously have a direct access to multiple smartphones’ local time clocks from the outside. Therefore, an experiment is designed to indirectly assess the time synchronization accuracy, which is explained as follows.

**Figure 9:**
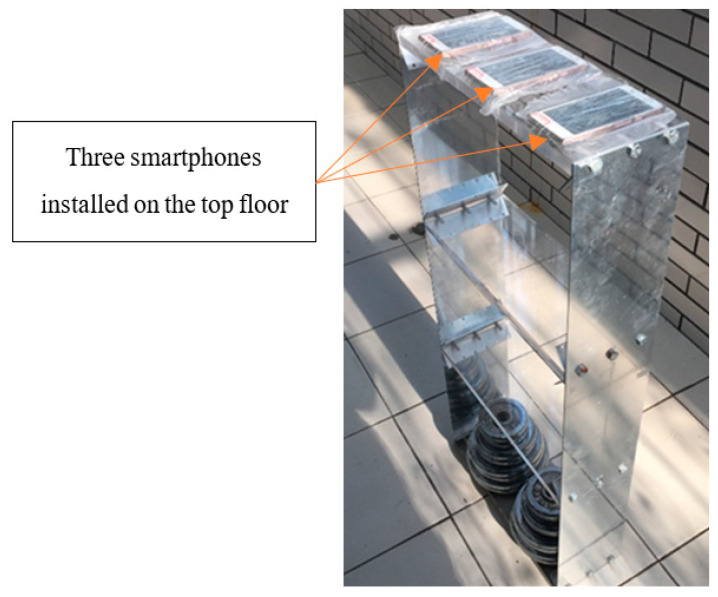
Time synchronization experimental setup.

Figure 9 shows the experimental setup. Three Huawei P6 smartphones, installing the iSHM-C, were attached to the top floor of a three-story scaled structural model as acceleration sensors. Another smartphone, installing the iSHM-S, is used as the server to remotely control these sensor smartphones. First, the time differences of the local time clocks between the server smartphone and the three sensor smartphones are estimated using the method proposed in Section 3. After the above synchronization operation is finished, the tested structure is pulled at the third floor and then suddenly released; the structural free-decayed responses are measured by the three sensor smartphones simultaneously. Since the three sensor smartphones measure the same structural response, if the local time clocks of these smartphones are accurately synchronized, the three structural responses measured these smartphones should match exactly in time domain.

### 4.2. Preprocess of Measured Structural Responses by Smartphones

**Figure 10:**
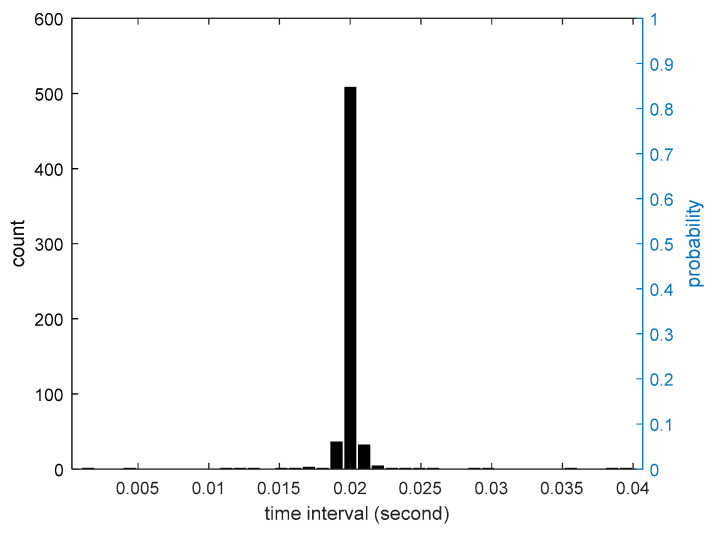
The distribution of time interval of response data collected by smartphones.

**Figure 11:**
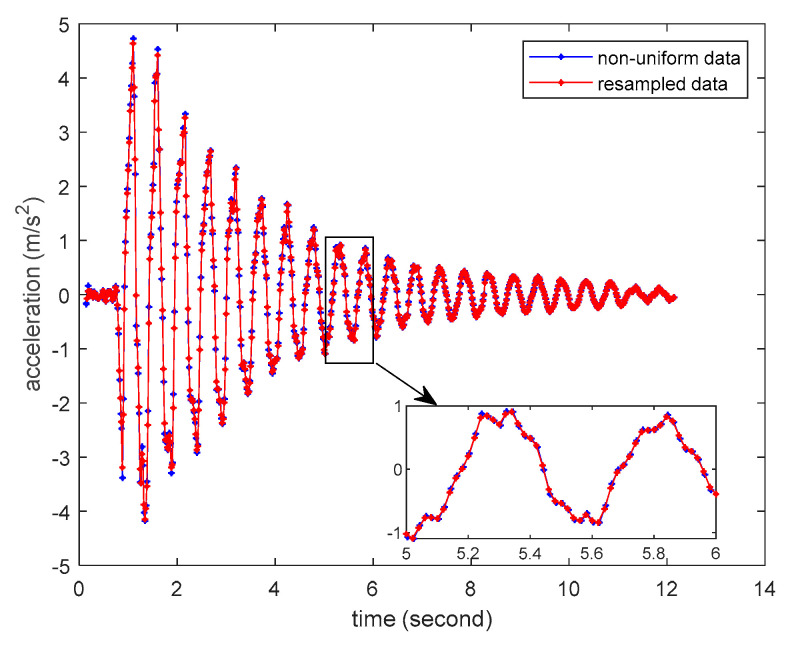
Comparison of measured non-uniformly distributed structural response and resampled uniformly distributed structural response.

Since smartphones are not initially designed for structural vibration monitoring, the developed smartphone-based system can only rely on Android system built-in function “onSensorChanged” to access the vibration sensor’s data in the smartphone. However, such approach cannot guarantee that the smartphones collect the vibration data at a pre-defined fixed time interval. Figure 10 shows the distribution the time intervals of the two adjacent measurements collected by the test smartphones. It can be seen that most of time intervals of the collected data are around 0.02 s, but there are a few cases that the time intervals are much smaller or larger. Therefore, the structural responses measured by smartphones are not uniformly distributed at a fixed time interval, which poses a difficulty for consequent data analysis. To cope with this problem, a simple linear interpolation is adopted in this study to resample the non-uniformly distributed structural responses measured by the smartphone to a uniformly distributed time axis with the fixed time interval of 0.02 s. Figure 11 shows the comparison of a non-uniformly distributed structural response directly measured by the smartphone and a uniformly distributed resampled response, demonstrating that the resampled uniformly distributed response can very accurately capture the dynamic characteristics of the original non-uniformly distributed response.

### 4.3. Experimental Results of Smartphones’ Time Synchronization

**Figure 12:**
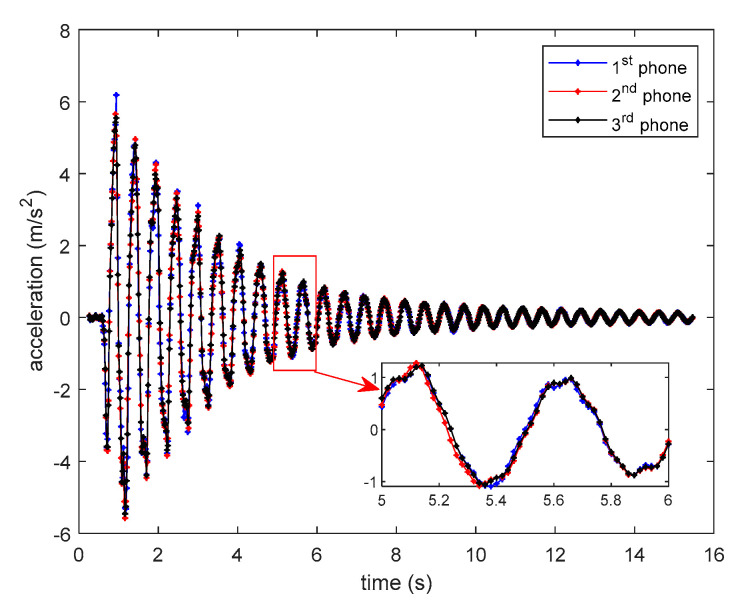
Smartphones’ measured structural responses.

Because the three smartphones measure the same structural response, if the phones are accurately time-synchronized, the three recorded structural responses should be well overlap in time domain. Figure 12 shows the structural free-decayed responses measured by the three sensor smartphones with the synchronized time clock, from which it can be clearly seen that all three structural responses almost exactly overlap, indicating that the proposed method have achieved very high accuracy of time synchronization. However, Figure 11 does not provide a quantitative time synchronization error. To quantitively evaluate the time synchronization accuracy, the following method is adopted. Note that the structural responses measured by the three testing smartphones cannot be the same, the synchronization index is defined as in Equation (4):

(4) $$J\left(\tau \right)=\frac{\sqrt{\sum_{i=1}^{N}{\left[x\left(i\Delta t\right)-y\left(i\Delta t+\tau \right)\right]}^{2}}}{\sqrt{\sum_{i=1}^{N}{\left|x\left(i\Delta t\right)\right|}^{2}}}$$

where x(iΔt) and y(iΔt) denote the structural responses of two sensor smartphones at the discrete time iΔt, and Δt is the time interval of measured structural responses, τ is the time shift of the two measured responses. When the index J(τ) takes the smallest value, we consider the corresponding time shift τ as the time synchronization error between the two sensor smartphones.

**Figure 13:**
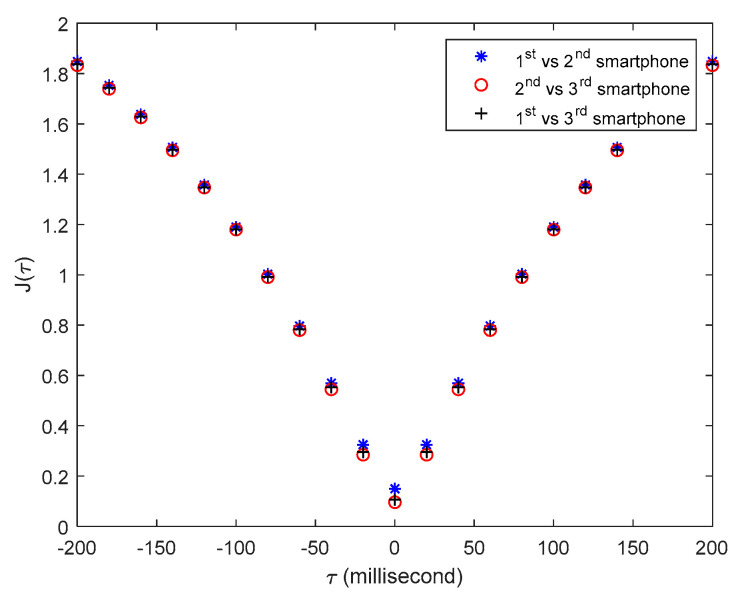
Time synchronization indexes J(τ) among there tested smartphones.

Figure 13 shows the values of the time synchronization indices J(τ), calculated by using the synchronized structural responses from any two of the three sensor smartphones. It is clear that all indices J(τ) have the smallest values at time shift τ=0, and at the adjacent time shift points (i.e., τ=±20 millisecond) the indices’ values are much larger than those at τ=0, indicating that the time synchronization error of the designed smartphone-based system is less than 20 milliseconds.

### 4.4. Time Synchronization Accuracy Analsys with Upsampled Structural Response

**Figure 14:**
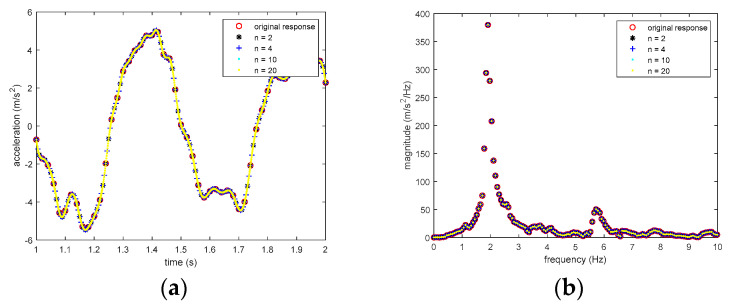
Comparison of original and upsampled structural response with different upsampling rates: (**a**) time domain response; (**b**) frequency domain response.

Due to the hardware limitation, the smallest time interval of the measured responses that the smartphones in this test can reach is only 0.02 s (i.e., 20 milliseconds), and no structural responses are recorded between two adjacent discrete time steps. Since, in the proposed time synchronization accuracy analysis method, the smallest of the time shift τ is one sample, the resolution of the analysis is limited by the time interval of the measured responses (i.e., 20 milliseconds). To improve the resolution of the time synchronization analysis, the following method is adopted. Noting that the structural free-decay responses in the test only contain the vibration of low frequencies, the structural responses between the two discrete time steps can be obtained by upsampling the measured responses via polyphase antialiasing filter [18] without introducing additional errors. Therefore, if the measured structural responses are upsampled *n* times, then the time interval of the structural responses can be reduced to 20/*n* milliseconds; and the resolution of the time synchronization analysis is also improved to 20/*n* milliseconds. Note that an antialiasing FIR lowpass filter of order 20*n is utilized in the above upsampling procedure. Figure 14 shows the comparison of the original and upsampled structural responses with different upsampling rates from one smartphone, demonstrating that the original and upsampled structural response perfectly match at the original measured points in both time and frequency domains as expected.

Moreover, it needs pointing out that the above upsampling operation does have some limitations. Because the upsampled responses can only match the underlining continuous responses given that the sampling time instances and the magnitudes of the sampled response are perfectly accurate, which is not true in practice. The resolution of the sampling time instance in the smartphone is limited to 1 millisecond, and the measured response is not accurate either due to the sensor’s imperfection. Both of them determine that the proposed upsampling method cannot accurately reconstruct the underlining continuous structural response. With a higher upsampling rate, more artificially generated response data points will be added to the original response, which may induce higher uncertainty of the calculated index J(τ) and make the corresponding time synchronization error more likely to be inaccurate.

To cope with the above problem, an adaptive upsampling strategy is adopted herein. A series of upsampling operations with upsampling rate from small to large are conducted and the time synchronization errors in every upsampled response are calculated based on the smallest value of the index J(τ). Then, the finial time synchronization error is determined by the largest value among all calculated errors. The above proposed adaptive upsampling strategy provides a new to improve the resolution of time synchronization accuracy analysis with reasonably reliable results, which avoid the inaccurate results from the operations with very high upsampling rates. If an operation with a lower upsampling rate gives a larger time synchronization error than the result of another operation with a higher upsampling rate, it will be reasonable to accept the result from the low upsampling rate operation, which uses less artificially generated data points.

**Figure 15:**
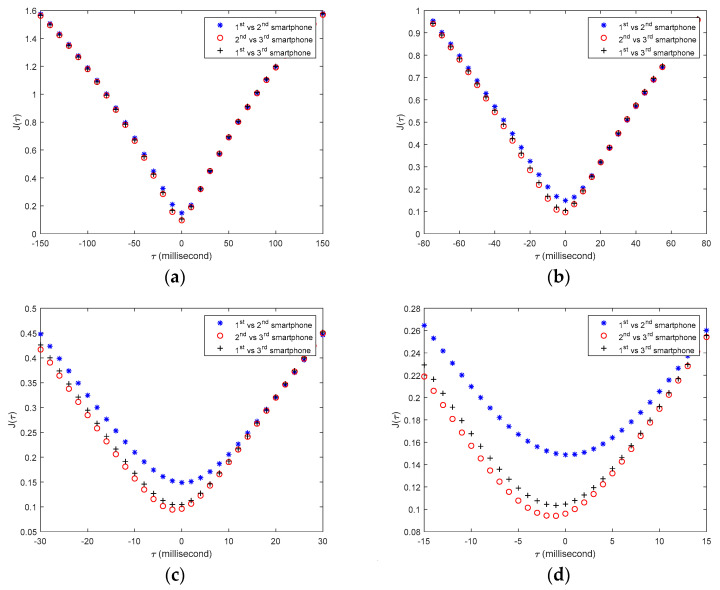
Time synchronization indices J(τ) calculated from up-sampled structural response with different time intervals: (**a**) *n* = 2, dt = 0.01 s; (**b**) *n* = 4, dt = 0.005 s; (**c**) *n* = 10, dt = 0.002 s; (**d**) *n* = 20, dt = 0.001 s.

Using the proposed method, the upsampled structural responses and the corresponding time synchronization indices among the three tested sensor smartphones are re-calculated. Four upsampling rates (i.e., *n* = 2, 4, 10, 20) are used in this study. Figure 15 shows the values of the time synchronization indices calculated from one upsampled structural response with different upsampling rates. Table 2 lists the calculated time synchronization errors with different upsampling rates, the final time synchronization errors are highlighted with yellow color.

In this study, totally four time-synchronization tests are conducted with the same structure. Table 3 gives the time synchronization errors between any two sensor smartphones in all the tests, which are obtained from the proposed adaptive upsampling procedure. It can be seen that the largest time synchronize error between any two sensor smartphones in all tests is only 5 milliseconds, verifying that the time synchronization method proposed in Section 3 can accomplish the millisecond-level time synchronization for the developed smartphone-based wireless structural vibration monitoring system.

### 4.5. Additionl Time Synchronization Tests

**Figure 16:**
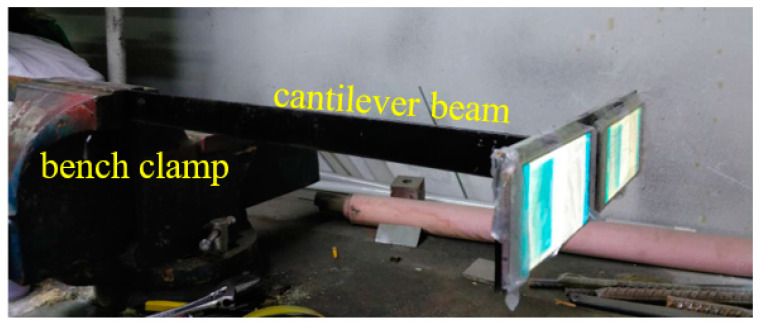
Setup of test.

To further verify the practical feasibility of the proposed time synchronization test procedure, another test is conducted here. In this test, a cantilever beam is used, as shown in Figure 16. Two smartphones of different models are attached to the free end of the beam. The other end of the beam is firmly clamped to a bench clamp. The first natural frequency of the cantilever beam can be adjusted via changing the clamp position of on the beam. In this test, five scenarios of different clamped positions are considered, which are corresponding to the beam’s length of 60, 50, 40, 30 and 20 cm, respectively. The corresponding first natural frequencies of the cantilever beams are identified to be 4.2, 5.4, 7.3, 9.8 and 14.9 Hz, respectively. For each scenario, five time synchronization tests are conducted using the method proposed thereafter.

**Figure 17:**
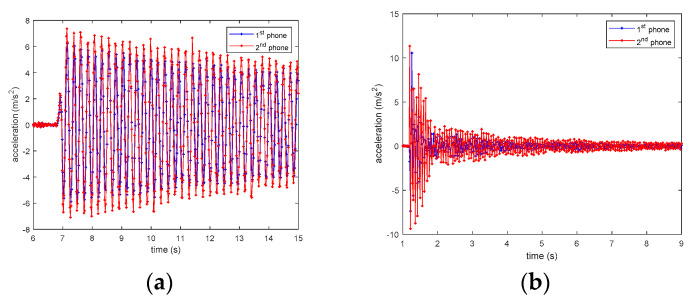
Smartphones’ measured structural responses in the test: (**a**) beam length = 60 cm; (**b**) beam length = 20 cm.

An important difference between the tests in this subsection and the previous subsection is that the two smartphones are of different model; therefore, these smartphones measure the same structural responses, the actual measurements will be different to some extent. Therefore, in this subsection, instead of using the synchronization index in Equation (4), the opposite value of cross correlation coefficient function $J_{1}\left(\tau \right)$, defined in Equation (5), is used to determine the time synchronization error. As shown in Figure 17, the free decay responses of the cantilever beams with lengths of 60 and 20 cm are significantly different. Because the 20 cm beam is very stiff, and its free decay response attenuates very fast.

(5) $$J_{1}\left(\tau \right)=-\frac{\sum_{i=1}^{N}x\left(i\Delta t\right)y\left(i\Delta t+\tau \right)}{\sqrt{\sum_{i=1}^{N}{\left|x\left(i\Delta t\right)\right|}^{2}}\sqrt{\sum_{i=1}^{N}{\left|y\left(i\Delta t\right)\right|}^{2}}}$$

**Figure 18:**
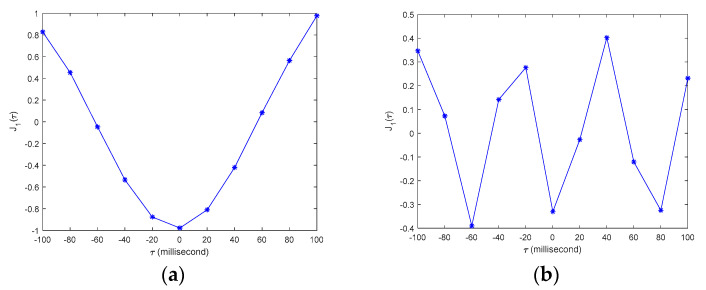
Time synchronization indexes $J_{1}\left(\tau \right)$ of cantilever beams: (**a**) beam length = 60 cm; (**b**) beam length = 20 cm.

Figure 18 shows the comparison of the indices $J_{1}\left(\tau \right)$ for the beams with the length of 60 and 20 cm, respectively, without the upsampling procedure proposed in Section 4.4. For the 60 cm beam, as the expected that $J_{1}\left(\tau \right)$ takes the minimum at τ=0; however, for the 20 cm beam, the smallest $J_{1}\left(\tau \right)$ occurs at τ=−60. This is due to the reason that the 20 cm beam’s response is a periodic response with an attenuating magnitude, and its corresponding index $J_{1}\left(\tau \right)$ should have periodic local minimal values. Moreover, the measured responses of the 20 cm beam has low quality as can be seen in Figure 17b; thus, the corresponding index $J_{1}\left(\tau \right)$ may not take its global minimal value at its true synchronized time instance. However, it needs pointing out that the time synchronization error of the designed smartphone-based system is determined after the time synchronization process, described in Section 3, is finished. This error will not change no matter what kinds of structures are used herein to check the time synchronization accuracy. According to the all previous testing results, it is very unlikely that the time synchronization error is larger than the time interval of the measured response (i.e., 0.02 s). Therefore, when facing with very stiff structure, an additional constraint that limits the searching domain of the minimal $J_{1}\left(\tau \right)$ to two time intervals of the measurement (i.e., 0.04s) can be added to ensure that the proposed method can find the true time synchronization error.

**Figure 19:**
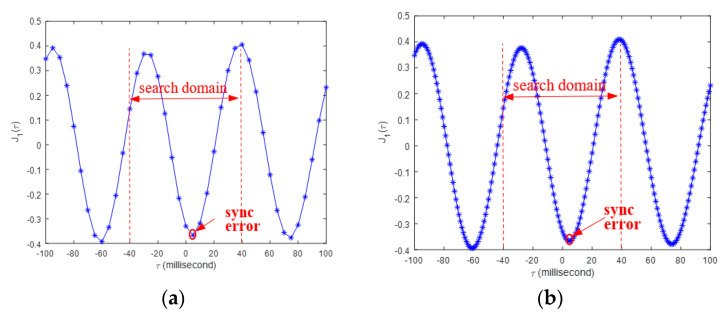
Time synchronization indexes $J_{1}\left(\tau \right)$ of the 20 cm beam with different upsampling rates: (**a**) *n* = 4 dt = 0.005 s; (**b**) *n* = 20 dt = 0.001 s.

With the addition constraint, the adaptive upsampling procedure, proposed in Section 4.4, is adopted to calculate the time synchronization errors of the cantilever beam tests in this subsection. Figure 19 shows the time synchronization indexes $J_{1}\left(\tau \right)$ of the 20 cm beam with two different upsampling rates, which demonstrates that if the addition constraint is included, the proposed method can still find the true synchronization error near the point τ=0. The time synchronization errors of all tests in this subsection are calculated and the results are given Table 4, from which it can be seen that the smartphone-based system can give quite accurate synchronization results, and most time synchronization errors are less than 10 milliseconds.

## 5. Experiments of Structural Modal Parameter Identification Using Smartphones

**Figure 20:**
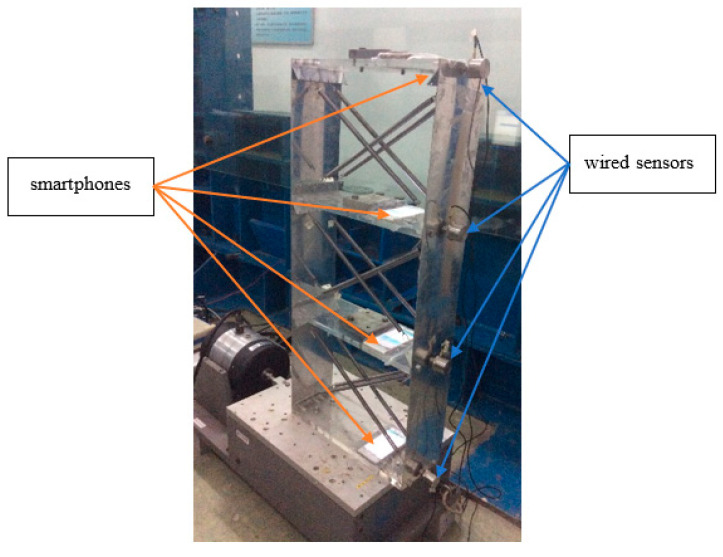
Setup of shake table test.

Structural modal parameter identification is one of most important applications for structural vibration monitoring. In order to evaluate the performance of the developed smartphone-based system for structural modal parameter identification, shake table tests are conducted on a 3-story bench-scale shear structural model, as shown in Figure 20. The test structure consists of two aluminum plates connected by four thick Plexiglas plates; four springs are added to each story of the structure to increase structural stiffness. Before the experiment, the test structure is dismantled to measure its mass and stiffness information. The information of floor mass and story stiffness of the test structure is given in Table 5.

The structure was shaken at the bottom. The structural accelerations of all floors are measured by the smartphones and the wired accelerometers simultaneously. Table 6 gives the specifications of the acceleration sensor in the smartphone and the wire accelerometer.

### 5.1. Frequency Domain Decomposition

To identify the structural natural frequencies and mode shapes, the frequency domain decomposition (FDD) method is adopted [19,20] herein, which is briefly reviewed in this subsection. It is assumed the structural excitation is a white-noise. Then, the power spectrum matrix $G_{yy}$ of the structural responses can be written as:

(6) $$G_{yy}\left(j\omega \right)=H\left(j\omega \right)\cdot C\cdot H{\left(j\omega \right)}^{H}$$

where *C* is the power spectrum of the structural excitation; superscript *H* denotes the complex conjugate transpose operation; H(jω) is the transfer function vector from the structural excitation to the structural responses, which can be written in partial decomposition format as:

(7) $$H\left(j\omega \right)=\overset{N}{\underset{k=1}{\sum }}\left(\frac{\phi_{k}r_{k}^{T}}{j\omega -\lambda_{k}}+\frac{\bar{\phi_{k}r_{k}^{T}}}{j\omega -\bar{\lambda_{k}}}\right)$$

where $\phi_{k}$ and $r_{k}$ are the mode shape and modal participation factor of the *k*-th mode; overhead bar denotes complex conjugate operation. Combining Equations (6) and (7), the power spectrum matrix $G_{yy}$ can be written as:

(8) $$G_{yy}\left(j\omega \right)=\overset{N}{\underset{k=1}{\sum }}\left(\frac{A_{k}}{j\omega -\lambda_{k}}+\frac{A_{k}^{H}}{j\omega -\bar{\lambda_{k}}}+\frac{A_{k}^{*}}{-j\omega -\lambda_{k}}+\frac{A_{k}^{T}}{-j\omega -\bar{\lambda_{k}}}\right)$$

where $A_{k}$ is the *k*^th^ residue of matrix $G_{yy}$ and can be calculated as:

(9) $$A_{k}=\phi_{k}r_{k}^{T}\cdot C\cdot r_{k}\phi_{k}^{T}=d_{k}\phi_{k}\phi_{k}^{T}$$

where $d_{k}=r_{k}^{T}\cdot C\cdot r_{k}$.

Since the structural damping ratios are small for general civil structures, $G_{yy}$ has sharp peaks in magnitude near the structural natural frequencies. Thus, the structural natural frequencies can be easily identified by the simple peak-pick method. After the structural natural frequencies are identified, the following procedure can be applied to estimate structural mode shapes. Since the structural power spectrum responses near certain structural natural frequency is dominated by the corresponding modal response, the value of power spectrum matrix $G_{yy}\left(j\omega \right)$ at the *k*-th structural natural frequency $\omega_{k}$ can be approximated by [21].

(10) Gyy(jωk)≈ϕk[diag(2Re(dkjωk−λk))]ϕkT

Then, the singular value decomposition of matrix $G_{yy}\left(j\omega_{k}\right)$ can be carried out. Based on the result of Equation (10), the *k*-th mode shape of the structure can be approximated by the 1st singular value vector of the singular value decomposition.

### 5.2. Identification Results of Structural Modal Parameters

#### 5.2.1. Natural Frequencies

Totally, five tests are conducted in this study. In each test, 10 min structural responses are measured by the smartphones and the wired sensors. The sampling rates for the smartphone sensors and wired sensors are 50 and 200 Hz, respectively. The power spectra of all structural responses are calculated via the Welch method [22]. To reduce the estimation error of the power spectra due to the power leakage, a Hanning window with the length of 20.48 s is used to preprocess the data.

**Figure 21:**
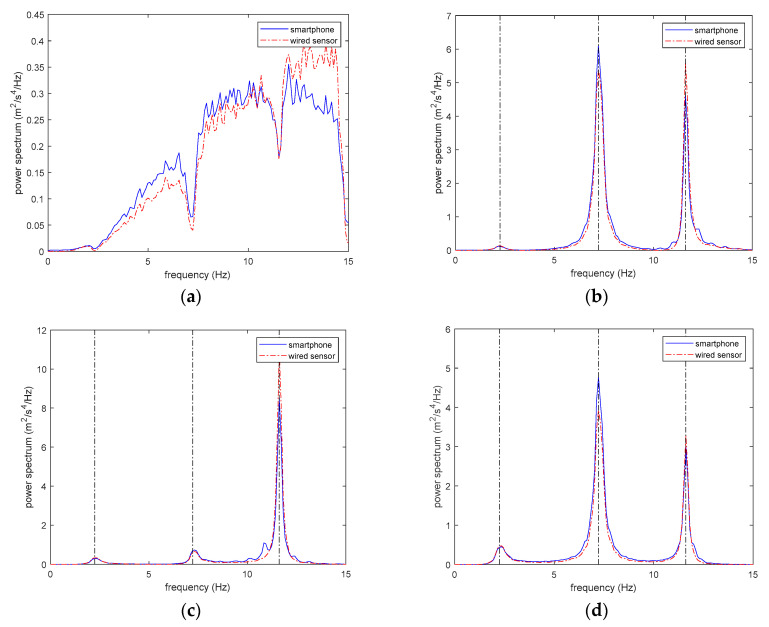
Comparison of power spectra of structural accelerations measured by smartphones and wired sensors: (**a**) base excitation; (**b**) 1st floor acceleration; (**c**) 2nd floor acceleration; (**d**) 3rd floor acceleration.

Figure 21 shows the estimated power spectra of structural responses from the smartphones and the wired sensors respectively, from which it can be seen that the power spectra estimated from the smartphones match those from the wired sensors well with some small discrepancy. This result is not surprising, because the MEMS acceleration sensors in the smartphones are not designed for accurate vibration measurement. Although the structural accelerations measured by the smartphones are not as accurate as professional wired sensors, the structural natural frequencies can still be estimated quite accurately. As shown in Figure 21b–d, the peak frequencies of the power spectra from the smartphones and the wired sensors match almost exactly, indicating that the structural natural frequencies estimated by the smartphones and the wired sensors will be almost same.

Table 7 and Table 8 list the estimate structural natural frequencies by the smartphones and the wired sensors, respectively. It can be seen that both smartphone sensors and wired sensors provide quite accurate estimation results. If the mean estimates of structural frequencies of the smartphone sensors and the wired sensors are compared, the largest relative error of the identified frequencies will only be 1.3%. The reason that the structural frequencies can be quite accurately estimated by inaccurate measurements from the smartphones is that the identification accuracy of structural frequencies is only related to the frequency locations of the peak responses of the power spectra and not the magnitudes of the peak responses.

#### 5.2.2. Mode Shapes

**Figure 22:**
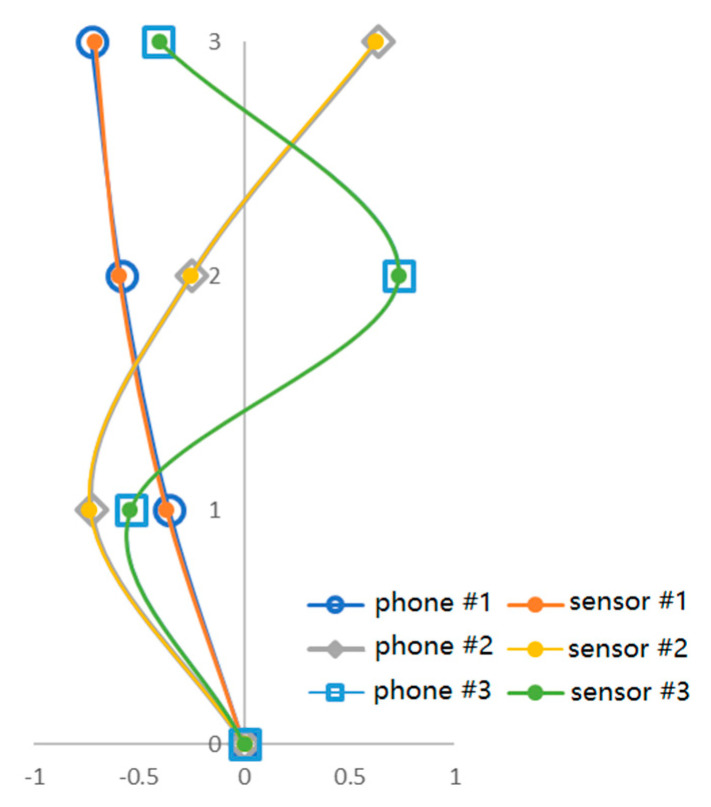
Comparison of estimated mode shapes.

After the structural natural frequencies are identified, the power spectrum matrices of structural responses at the structural natural frequencies are extracted. The singular value decomposition operation is conducted for the power spectrum matrices to estimate the structural mode shapes. Figure 22 shows the comparison of the estimated mode shapes in one test, which almost exactly match each other.

To quantitively evaluate the accuracy of the identified mode shapes, the mode assurance criterion (MAC) value of the estimated mode shapes by the smartphones and by the wired accelerometers are computed. As demonstrated in Equation (11), the MAC value reflects the linear similarity of two mode shape vectors, which takes the value between zero and one. The MAC value being very close to one indicates that the two mode shapes are almost same.

(11) $$\mathrm{MAC}=\frac{\left|\phi^{T}\varphi \right|}{\sqrt{\left|\phi^{T}\phi \right|\cdot \left|\varphi^{T}\varphi \right|}}$$

where ϕ and φ denote the two identified mode shape vectors. It is noted that the MAC value is only a good mode shape accuracy indicator for structures with a few DOFs, as for the structure with many DOFs, the MAC value becomes insensitive to the changes of the mode shapes.

Table 9 lists the MAC values of the estimated mode shapes by the smartphones and the wired sensors. The MAC values of all three estimated mode shapes are very close to one, verifying that the mode shapes estimated by the smartphones are close to those estimated by the wired sensors. As can be seen in Figure 21b–d, there are observable differences of the peak magnitudes of the spectra obtained from the smartphones and the wired sensors, which are probably due to the inaccuracy of the smartphone integrated sensors. Since the accuracy of the identified mode shapes by the FDD method is directly related to the magnitude of the peak responses in the spectrum responses, how not very accurate structural spectrum responses can obtain relatively accurate identification results of mode shapes is kind of interesting.

This interesting phenomenon may be explained as follows. Although the sensors integrated in the smartphones are not accurately calibrated as the professional wired sensors, all the smartphones used in the tests belong to the same model (i.e., Huawei P6) and it is very likely that these vibration sensors are very similar. Therefore, the peak magnitudes of the spectrum responses measured by all the smartphones can be considered to be amplified or reduced by a similar factor. According to the nature of the FDD method, if the power spectrum matrix $G_{yy}\left(j\omega \right)$ is only multiplied by a factor, the identified mode shapes will not be changed. This may explain why the mode shapes identified by the smartphones are quite accurate, even though the structural accelerations measured by the smartphones are not that accurate.

### 5.3. Modal Parameter Identification of Damaged Structure

**Figure 23:**
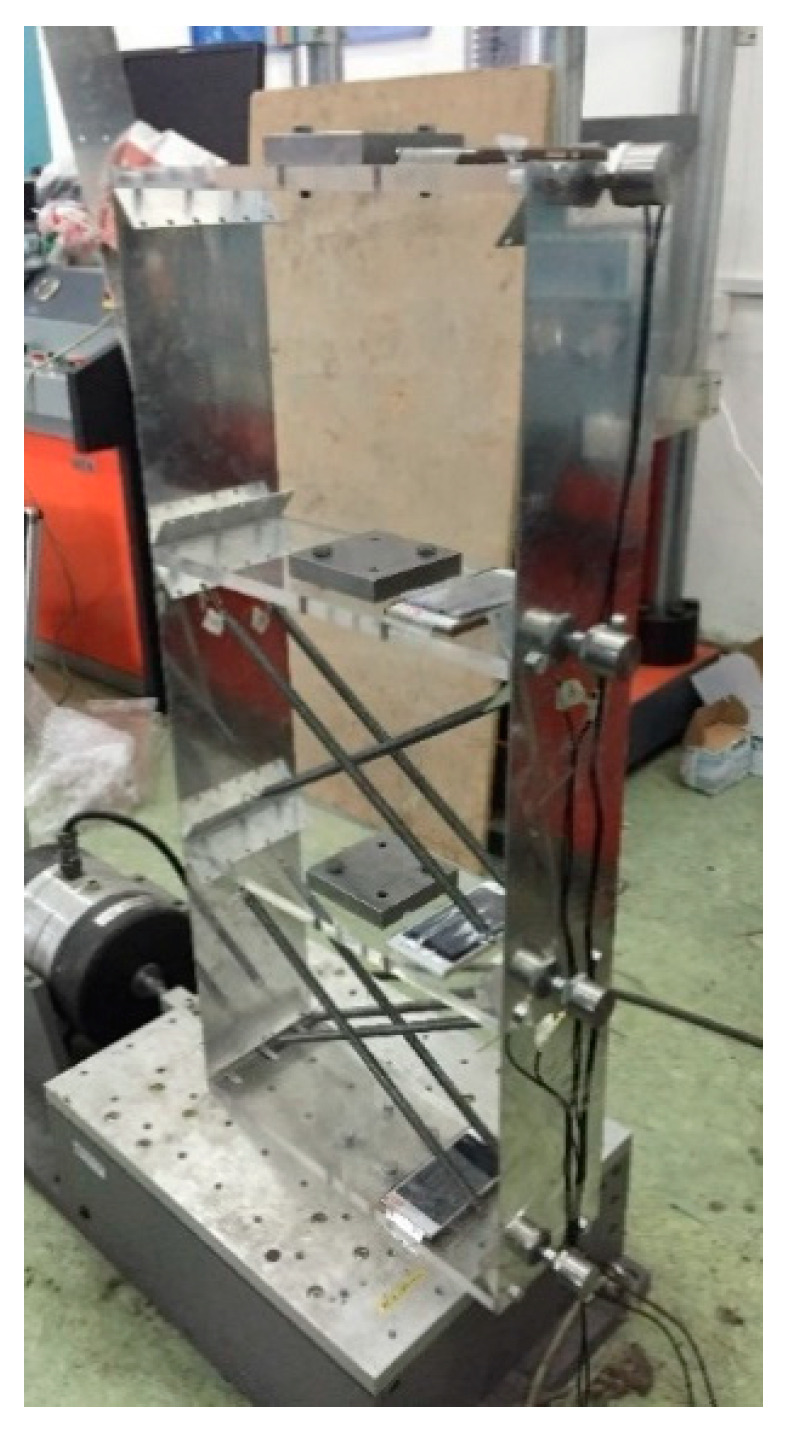
Damaged structure of the 1st scenario.

To further test the ability of the developed smartphone-based vibration monitoring system, two damage scenarios were simulated by removing some diagonal springs of the test structure. In the first scenario, the four springs of the 3rd story were removed. In the second scenario, the four springs of 2nd story were removed. The damage condition is proximately equal to reducing the corresponding stiffness by about 20%. Figure 23 gives the picture of the damaged structure of the 1st scenario. The structural natural frequencies and mode shapes were identified by using the acceleration responses of both damaged structures, measured by the smartphones and wired sensors respectively. For each damaged scenario, five tests are conducted, each of which utilizes the 10-min structural responses. The mean values of the estimated natural frequencies by the smartphones and the wired sensors are compared in Table 10. The MAC values of the mean value of the estimated mode shapes between the smartphones and wired sensors are calculated shown in Table 10. The results in Table 10 and Table 11 verify again the feasibility of the developed system for structural modal parameter identification.

### 5.4. Discussion

It is worth of mentioning that although the experimental results in this paper demonstrated that the developed system can achieve quite accurate structural modal identification results, it does not provide any implication of the identification accuracy of future experiments using different smartphones, as the developed software is based on the android system. Theoretically, it can be used on any android smartphone. However, the structural identification accuracy highly depends on the accuracy of measured structural responses, which relies on quality of the integrated vibration sensors in the tested smartphone. Different smartphones may have different integrated vibration sensors which have different measurement accuracy. Therefore, in order to have accurate structural identification results using the developed software in this paper, the first crucial step is to select the smartphones that integrate high quality vibration sensor, which is out of control of the developed software.

## 6. Conclusions

In this study, an android-smartphone based structural vibration system was developed, which can easily convert multiple android smartphones into a wireless sensor network to measure structural vibration. The server/client architecture is adopted for the designed system. One smartphone is selected as the server to remotely control all other smartphones that serves as sensors to measure structural accelerations. A new method is proposed to quickly set up the network communication between the server and sensor smartphones, which facilitates fast deployment of a lot of smartphones as sensors on the structure. Furthermore, to ensure sensor smartphones simultaneously measuring structural responses, a time synchronization method is proposed for the smartphone-based structural vibration monitoring system, which is verified by the experiment to be able to achieve the time synchronization accuracy of millisecond level between sensor smartphones. Finally, a shaking table experiment is conducted, which demonstrates that the designed smartphone-based wireless structural vibration monitoring system can quite accurately identify the modal parameters of the tested structure.

Although the preliminary study in this paper has demonstrated promising potentials for the developed smartphone-based structural vibration monitoring system, more comprehensive tests are still needed to further test the performance of the developed system in more complex working environments. Because the performance of the time synchronization and the structural modal parameter identification of the developed system is affected by many parameters, including the specifications of the smartphone sensors, the electro-magnetic working environment of the system, etc., all of these have not been extensively tested in this paper, which will be an important research direction in future.

## Figures and Tables

**Table 1:** Technical parameters of smartphone-integrated wireless communication technologies.

Technical Parameters	Smartphone-Integrated Wireless Communication Technologies			
	4G	WIFI	Bluetooth	NFC
number of connected devices	multiple	multiple	multiple	single
connection distance	no limit	~100 m	~10 m	~0.2 m
data transmission bandwidth	100 Mbps	54 Mbps	2.1 Mbps	424 kbps
cost to use	Not free	free	free	free

Note: most information in this table are obtained from the website of www.wikipedia.org.

**Table 2:** Time synchronization errors with different upsampling rates (millisecond).

Upsampling Rate *n*	1st Phone vs.2nd Phone	2nd Phone vs. 3rd Phone	1st Phone vs. 3rd Phone
2	0	0	0
4	0	0	0
10	0	−2	−2
20	0	−1	−1

**Table 3:** Time synchronization errors between any two sensor smartphones (millisecond).

Test #	1st Phone vs. 2nd Phone	2nd Phone vs. 3rd Phone	1st Phone vs. 3rd Phone
1	0	−2	−2
2	1	−2	−2
3	−1	−2	−5
4	1	−1	0

**Table 4:** Time synchronization errors of cantilever beams (millisecond).

Beam Type	Test #				
	1	2	3	4	5
20 cm	5	10	6	5	10
30 cm	−10	−10	−8	−8	−10
40 cm	−6	−6	−6	−8	−6
50 cm	−5	−5	−5	−5	−5
60 cm	−5	−5	−2	−2	−3

**Table 5:** Floor mass and story stiffness of test structure.

Parameters	Story #		
	1	2	3
Mass (g)	3720.2	3720.2	3265.2
Stiffness (N/m)	4533	4533	4533

**Table 6:** Technical specifications of smartphone vibration sensor and wired accelerometer.

Sensor Model	Measurement Range	Sensitivity	Noise Level	Frequency Range (Hz)	Operation Temperature (°C)
LIS303DLHC (smartphone)	±40 m/s^2^	18 mg/digit	±0.03 m/s^2^	0~100	−40~85
KD1100 (wired sensor)	±20 m/s^2^	1000 pC/g	n/a	0.2~2.5k	−20~100

**Table 7:** Estimated structural frequencies by smartphones (Hz).

Mode #	Test #					Mean
	1	2	3	4	5	
1	2.344	2.295	2.246	2.295	2.246	2.285
2	7.227	7.227	7.275	7.275	7.275	7.256
3	11.621	11.670	11.670	11.670	11.670	11.660

**Table 8:** Estimated structural frequencies by wired sensor (Hz).

Mode #	Test #					Mean
	1	2	3	4	5	
1	2.344	2.295	2.246	2.344	2.344	2.315
2	7.227	7.227	7.275	7.275	7.275	7.256
3	11.621	11.670	11.670	11.670	11.670	11.660

**Table 9:** MAC values of estimated mode shapes by smartphones and wired accelerometers.

Mode #	Test #				
	1	2	3	4	5
1	0.9996	0.9995	0.9995	0.9990	0.9986
2	0.9997	0.9997	0.9998	0.9996	0.9999
3	0.9994	0.9999	0.9999	0.9997	0.9999

**Table 10:** Mean estimates of damaged structures by smartphones and wired accelerometers (Hz).

Mode #	1st Scenario		2nd Scenario	
	Smartphone	Wired Sensor	Smartphone	Wired Sensor
1	2.285	2.256	2.324	2.315
2	7.324	7.324	7.168	7.139
3	11.533	11.523	11.621	11.529

**Table 11:** Mode assurance criterion (MAC) values of estimated mode shapes of damaged structure by smartphones and wired accelerometers.

Mode #	1st Scenario	2nd Scenario
1	0.9996	0.9998
2	0.9998	0.9998
3	0.9998	0.9999
